# Lack of Significant Associations Between Hematological Parameters, Salivary Gland Histopathology and Fatigue in Sjögren’s Disease

**DOI:** 10.3390/ijms262311418

**Published:** 2025-11-26

**Authors:** Denise-Ani Mardale, Mihai Alexandru Preda, Daniela Opriș-Belinski, Violeta Bojincă, Cristian-Mihai Ilie, Florian Berghea, Laura Maria Groșeanu, Andra Rodica Bălănescu

**Affiliations:** 1 Faculty of Medicine, ‘Carol Davila’ University of Medicine and Pharmacy, 050474 Bucharest, Romania; denise-ani.mardale@drd.umfcd.ro (D.-A.M.); daniela.opris@umfcd.ro (D.O.-B.); violeta.bojinca@umfcd.ro (V.B.); florian.berghea@umfcd.ro (F.B.); maria.groseanu@umfcd.ro (L.M.G.); andra.balanescu@umfcd.ro (A.R.B.); 2 Department of Internal Medicine and Rheumatology, ‘Sf. Maria’ Clinical Hospital, 011192 Bucharest, Romania; 3 Ear, Nose and Throat (ENT) Department, ‘Sf. Maria’ Clinical Hospital, 011192 Bucharest, Romania

**Keywords:** Sjögren’s disease, fatigue, hematological indices, immunologic markers, minor salivary gland biopsy

## Abstract

Fatigue is a common and disabling symptom in Sjögren’s disease (SjD), yet its links with hematologic parameters and salivary gland histopathology are not well established. This study aimed to investigate associations between complete blood count (CBC) indices, fatigue severity, and minor salivary gland biopsy findings in SjD using a multidimensional, disease-specific fatigue instrument. Ninety-seven patients meeting the 2012 ACR criteria for SjD underwent CBC, immunologic testing, and detailed clinical evaluation. Fatigue was assessed with the Profile of Fatigue and Discomfort–Sicca Symptoms Inventory (PROFAD-SSI-SF). Histopathology data, including focus score, were available for a subset of patients. Correlation analyses, subgroup comparisons, and multivariable regressions explored associations among hematologic, immunologic, and fatigue variables. No strong correlations were found between fatigue and hematologic indices. The strongest were between total PROFAD and leukocyte (r = 0.18) or platelet counts (r = 0.16). ANA and anti-Ro52 positivity were associated with higher total SSI scores, while anti-SSB positivity correlated with lower somatic fatigue. Joint pain showed a borderline association with increased somatic fatigue. Focus score and other histopathologic features did not correlate with fatigue domains. In conclusion, fatigue in SjD is multifactorial and only weakly related to hematologic indices, emphasizing the need for longitudinal biomarker studies integrating clinical and histopathologic data.

## 1. Introduction

The change in terminology from “Sjögren’s syndrome” to “Sjögren’s disease” reflects an international consensus to better capture the systemic, pathogenic, and clinically significant nature of the disorder, moving away from the implication of a vague or loosely defined symptom complex that the term “syndrome” conveys. The 2023 International Rome Consensus recommends “Sjögren disease” as the official nomenclature, emphasizing its status as a distinct autoimmune disease with well-defined immunopathology, including chronic lymphocytic infiltration of exocrine glands and systemic manifestations such as fatigue and hematological abnormalities. This change is particularly relevant in research linking complete blood cell count (CBC) parameters, clinical manifestations (notably fatigue), and minor salivary gland biopsy findings, as it underscores the need for precise disease characterization and stratification in both clinical and investigational settings [1].

Sjögren’s disease is a chronic, systemic autoimmune disorder characterized by lymphocytic infiltration and dysfunction of exocrine glands, most notably the salivary and lacrimal glands, resulting in dry mouth and dry eyes as its hallmark clinical features [2,3,4]. Laboratory evaluation frequently reveals abnormalities on the complete blood cell count, including lymphocytopenia—particularly of the CD4+ T cell subset—and, less commonly, mild anemia or other cytopenias [4]. Recent high-dimensional immunophenotyping studies have identified a distinct peripheral blood signature in primary Sjögren’s disease, marked by decreased numbers of CD4+ T cells, memory B cells, and plasmacytoid dendritic cells, alongside increased activated T cells and plasmablasts; these changes correlate with disease activity and glandular inflammation [5].

### 1.1. Complete Blood Cell Counts Alterations in Sjögren’s Disease

CBC parameters, especially lymphocyte counts, are clinically relevant not only for disease monitoring but also for risk stratification, as persistent lymphocytopenia is associated with an increased risk of B-cell lymphoma in this population. The integration of CBC findings with serologic and immunologic markers enhances diagnostic accuracy and informs prognosis in Sjögren’s disease [6,7,8].

A complete blood count is a fundamental laboratory test that quantifies the major cellular components of blood, including red blood cells (RBCs), white blood cells (WBCs), and platelets, along with indices such as hemoglobin, hematocrit, mean corpuscular volume (MCV), and red cell distribution width (RDW) [9,10]. Recently, additional indices derived from the relationships between these cellular components have been described and may provide useful information in the evaluation of certain pathologies.

Over time, several studies have described associations between the neutrophil-to-lymphocyte ratio (NLR) and clinical manifestations in patients with Sjögren’s disease. An elevated NLR has been correlated with increased disease activity and has also been proposed as a potential marker for predicting systemic involvement [11,12]. Specifically, NLR has been linked to the development of interstitial lung disease (ILD) in Sjögren’s disease, suggesting a role in the assessment of pulmonary risk [13]. Additionally, higher NLR values have been associated with cutaneous vasculitis, reflecting a more active inflammatory state. These findings highlight the potential utility of NLR as a simple, accessible biomarker that may aid in identifying patients at risk for systemic complications beyond glandular involvement [11,14,15].

Fatigue is a highly prevalent and clinically significant symptom in primary Sjögren’s disease, affecting up to 70% of patients and substantially impairing quality of life [2,16,17]. Its pathogenesis is multifactorial and appears to be more strongly linked to psychosocial factors—such as depression, neuroticism, sleep disturbances, and comorbid fibromyalgia—than to routine hematologic or inflammatory parameters [18,19,20].

Current evidence does not support a direct or independent association between fatigue and complete blood cell count abnormalities, including lymphocytopenia, anemia, or neutrophil to lymphocyte ratio, in primary Sjögren’s disease [18,21]. While indices such as NLR, platelet to lymphocyte ratio (PLR), and monocyte to lymphocyte ratio (MLR) may reflect systemic disease activity or risk of extraglandular manifestations, they do not predict or explain fatigue severity [14,18,22,23,24].

Importantly, eosinophils and basophils are not recognized in the current medical literature as contributors to the pathogenesis of Sjögren’s disease or to the development of fatigue. The immunopathology of Sjögren’s disease is characterized by lymphocytic infiltration, B cell hyperactivity, and autoantibody production, with no established role for eosinophils or basophils in disease mechanisms or symptomatology [16,18,25,26].

Although these cell types are not currently implicated, their potential relevance will be explored in this study in accordance with clinical manifestations and fatigue, recognizing the need for further research to clarify any novel associations.

### 1.2. Tools to Measure Fatigue in Sjögren’s Disease

Validated tools and questionnaires used to assess fatigue in patients with Sjögren’s disease include both disease-specific and generic instruments. In addition to the Profile of Fatigue and Discomfort–Sicca Symptoms Inventory (PROFAD-SSI) and the Medical Outcomes Study Short Form 36 (MOS SF-36), several other measures are frequently applied. The EULAR Sjögren’s Syndrome Patient Reported Index (ESSPRI) is a disease-specific tool that quantifies patient-reported fatigue, pain, and dryness on a 0–10 numerical scale and is widely used in both clinical trials and routine practice. The Functional Assessment of Chronic Illness Therapy–Fatigue (FACIT-F) is a generic, well-validated scale that evaluates the impact of fatigue on daily functioning and has been extensively employed in SJD research [27,28,29,30].

Other instruments occasionally used include the Visual Analogue Scale (VAS) for fatigue, a simple single-item measure, and the Hospital Anxiety and Depression Scale (HADS), which, although not specific to fatigue, is often applied to explore the relationship between fatigue and mood disturbances. The Comprehensive Pain Evaluation Questionnaire (CPEQ) has also been adapted in SJD cohorts to include fatigue domains [18,31,32].

The Profile of Fatigue and Discomfort–Sicca Symptoms Inventory (PROFAD-SSI) is a validated, disease-specific patient-reported outcome measure designed to assess the multidimensional symptom burden in Sjögren’s disease, with a particular focus on fatigue and sicca symptoms. The short form (PROFAD-SSI-SF) consists of 19 items that capture somatic and mental fatigue, pain, and dryness across multiple domains (oral, ocular, cutaneous, vaginal, nasal, and otic). Each item is scored by the patient, and domain scores can be calculated to quantify the severity and impact of these symptoms in daily life [27].

The PROFAD-SSI-SF has demonstrated strong internal consistency, test–retest reliability, and convergent validity with other established measures of fatigue and dryness, such as the FACIT-F and ESSPRI. It is sensitive to change and can distinguish between different levels of disease activity and patient-reported global health. In clinical and research settings, the PROFAD-SSI-SF is used to systematically evaluate the burden of fatigue and sicca symptoms, both of which are highly prevalent and disabling in Sjögren’s disease [27].

A Romanian version of the PROFAD-SSI-SF has been translated and culturally adapted following standardized forward–backward translation procedures, ensuring conceptual equivalence with the original English version. This version preserves the psychometric properties of the original tool and is suitable for use in Romanian-speaking patient populations, facilitating both clinical assessment and research comparability across countries [33].

Given its standardized and multidimensional assessment of fatigue, the PROFAD-SSI-SF offers a valuable instrument for future research exploring possible associations between fatigue and hematological parameters, such as blood cell counts or inflammatory markers, in primary Sjögren’s disease. Its comprehensive coverage of both somatic and mental fatigue domains makes it particularly suited for studies aiming to clarify the biological underpinnings of fatigue and to evaluate the impact of targeted interventions.

There are significant gaps in the medical literature regarding the direct association between complete blood cell count parameters, fatigue severity, and minor salivary gland biopsy findings in patients with Sjögren’s disease. Most studies have focused on the diagnostic and prognostic value of minor salivary gland biopsy—particularly the focus score and lymphoid composition—for disease classification and risk stratification, but have not systematically linked these histopathological findings to CBC-derived markers or to patient-reported fatigue severity [22,34,35,36,37].

While some research has explored the prognostic significance of lymphocytic foci composition for disease flare and severity, these studies have not concurrently evaluated CBC parameters or fatigue outcomes in the same cohorts. Similarly, although fatigue is recognized as a major clinical burden in Sjögren’s disease, its relationship to objective laboratory markers—including CBC indices such as neutrophil or lymphocyte counts, or derived ratios like NLR—remains poorly defined, with most evidence suggesting a lack of direct correlation [12,22]. Furthermore, there is a lack of integrated studies that simultaneously assess CBC parameters, validated fatigue instruments (such as ESSPRI or FACIT-F), and detailed minor salivary gland histopathology.

In summary, there is a notable lack of comprehensive, prospective studies examining the relationship between CBC parameters, fatigue severity, and minor salivary gland biopsy findings in Sjögren’s disease. Addressing this gap, the present study correlates CBC-derived markers—such as neutrophil-to-lymphocyte ratio and platelet-based indices—with fatigue severity measured by validated instruments, and with histopathological features of minor salivary gland biopsies, including focus score and lymphocytic infiltration. This integrated approach aims to clarify the interplay between systemic immune activation, subjective symptom burden, and local glandular immune-mediated damage in Sjögren’s disease.

## 2. Results

A total of 97 patients with primary Sjögren’s disease were included in the study. The demographic and clinical characteristics are summarized in Table 1. The mean age of the cohort was 57.18 ± 14.1 years, with a clear predominance of female patients (96.9%). Most participants were from urban areas (90%). The mean age at diagnosis was 51.1 ± 14.33 years, with an average diagnostic interval of 1.66 ± 3.51 years.

Regarding lifestyle factors, 14.3% of the patients were current smokers. The most frequently reported clinical manifestations were xerostomia and xerophthalmia (both 92.8%), followed by joint pain (68.4%). Neurological and pulmonary manifestations were present in 21.4% and 27.6% of patients, respectively.

At the time of blood sampling, 32 patients were treatment-naive, whereas the remaining patients were receiving systemic therapy. Hydroxychloroquine was the most frequently used medication (72.2%), followed by glucocorticoids (36.1%), azathioprine (13.4%), methotrexate (7.2%), and mycophenolate mofetil (7.2%). These agents are typically reserved for patients with systemic or organ-threatening manifestations, which aligns with the presence of neurological or pulmonary involvement in a proportion of the study population.

**Figure 1:**
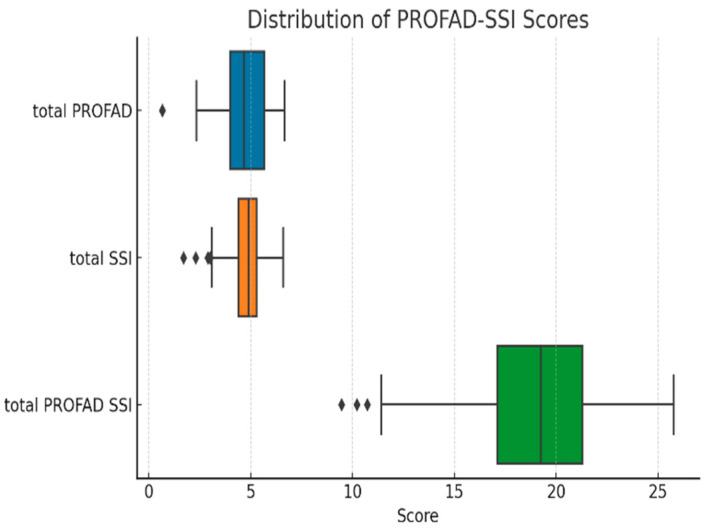
The distribution of fatigue and sicca symptom scores. Boxes represent the interquartile range (IQR), horizontal lines inside the boxes indicate the median, whiskers represent the minimum and maximum values within 1.5× IQR, and diamond symbols represent outliers.

Figure 1 illustrates the distribution of PROFAD, SSI, and total PROFAD-SSI scores in the study cohort. The median [IQR] scores were 4.67 [4.00–5.67] for the PROFAD domain, reflecting somatic and mental fatigue, 4.90 [4.40–5.30] for the SSI domain, which captures sicca-related symptoms, and 19.27 [17.10–21.30] for the combined PROFAD-SSI total score. While the SSI domain displayed the narrowest interquartile range, indicating relatively homogenous symptom severity across patients, the total PROFAD-SSI score showed greater variability. Outliers were identified in all domains, representing patients with unusually low fatigue or dryness scores, which may correspond to milder disease phenotypes or effective symptom control. These distributions suggest that, although fatigue and dryness burden are generally high in this cohort, a subset of patients experience substantially less symptom impact.

**Figure 2:**
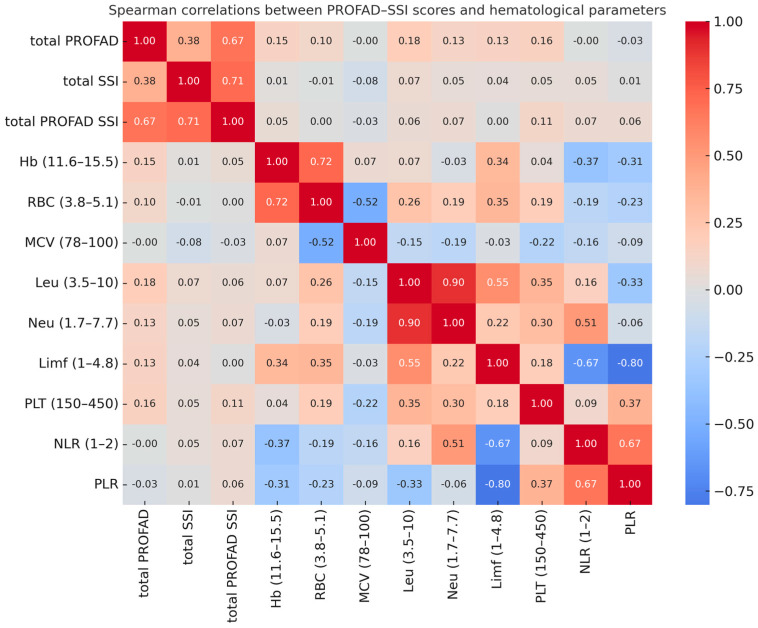
Spearman correlation analysis between PROFAD–SSI and Hematological parameters.

As shown by the Spearman correlation analysis (Figure 2), PROFAD, SSI, and total PROFAD-SSI fatigue scores showed only weak associations with hematological parameters, with no statistically significant correlations. The highest coefficients were observed between total PROFAD and leukocyte count (r = 0.18) and between total PROFAD and platelet count (r = 0.16), but these were of small magnitude and without clinical relevance. In contrast, strong correlations were found between interdependent hematological parameters, such as leukocyte and neutrophil counts (r = 0.90), neutrophil count and the NLR (r = 0.51), or lymphocyte count and the PLR (r = −0.80), reflecting internal consistency of the measurements rather than any association with fatigue.

As detailed in Table 2, none of the hematological parameters correlated significantly with fatigue scores, in line with the trends visualized in the heatmap (Figure 2). The highest coefficients with total PROFAD were seen for leukocyte count (r = 0.18), platelet count (r = 0.16), and hemoglobin (r = 0.15), indicating weak positive associations. Similar patterns were observed for total SSI and the combined PROFAD–SSI score, with platelet count showing the most consistent trend across all measures. While these associations did not reach statistical significance, they identify potential variables of interest for future studies in larger cohorts or in multivariable frameworks including inflammatory and immunological parameters.

These findings, although not statistically significant, support the continued investigation of hematological parameters—particularly leukocyte and platelet count—as potential contributors to fatigue severity in Sjögren’s disease, alongside salivary gland histopathology and immunological markers.

To derive the condensed summary presented in Table 3, we first conducted comprehensive multivariable linear regression models for hematologic, immunologic, and inflammatory parameters in relation to both somatic fatigue and total SSI scores. In parallel, subgroup comparisons (Mann–Whitney U tests) were performed for key clinical and serologic features, and non-parametric tests (Kruskal–Wallis) were used to assess trends across tertiles of NLR and PLR. All analyses were initially exploratory, with variables showing *p*-values < 0.10 considered for further inclusion. In the final condensed table, we report only those predictors with statistically significant or borderline associations (*p* < 0.05 or *p* < 0.10) to highlight the most relevant findings. Based on this multi-step analytical approach, the variables that remained significant or borderline significant were summarized in a condensed table to facilitate interpretation of the most relevant associations.

Minor salivary gland biopsies were available in 67 of the 97 patients (69.1%). Among these, 46 patients (47.4%) had a positive focus score (FS ≥ 1), whereas 21 (21.6%) had a negative biopsy (FS = 0). The median FS was 1. Additional quantitative histopathological parameters included number of foci, adipose score, and acinar atrophy score.

Correlation analysis showed that focus score did not correlate with PROFAD (r = −0.09, *p* = 0.67) or with total PROFAD-SSI (r = −0.36, *p* = 0.093). A moderate inverse association was found between focus score and SSI (r = −0.48, *p* = 0.021). This association indicated that higher focus scores were not accompanied by greater subjective sicca burden. Other histopathological variables did not show significant associations with PROFAD, SSI or total PROFAD-SSI scores (Table 4).

**Figure 3:**
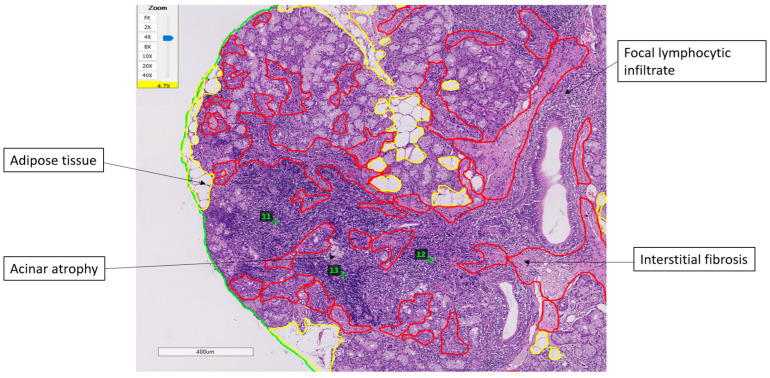
Representative minor salivary gland biopsy (H&E, 4.7×) illustrating the digital evaluation method and key histopathological features. The total glandular surface is outlined in green, adipose tissue is marked in yellow, and interstitial fibrosis is delineated in red. Lymphocytic infiltrates forming discrete foci (numbers) are also identified within the parenchyma. Acinar atrophy is visible in areas adjacent to the infiltrates.

Representative histopathological findings and the digital assessment method are illustrated in Figure 3. Minor salivary gland biopsies showed focal lymphocytic infiltrates forming well-defined foci, together with interstitial fibrosis, acinar atrophy, and areas of adipose tissue replacement. The total glandular surface, adipose compartments, and fibrotic regions were digitally delineated to ensure standardized quantification. These structural changes varied across patients and did not demonstrate significant associations with fatigue or PROFAD-SSI scores.

**Figure 4:**
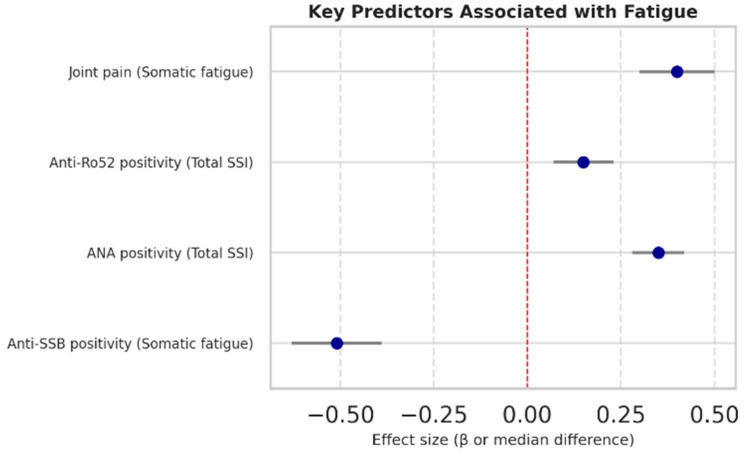
Forest plot of key predictors of fatigue outcomes in Sjögren’s disease. Effect sizes (β for linear models; median differences for non-parametric tests) with SEs are shown. The red dashed line represents the zero-effect reference (β = 0 or median difference = 0). Bullets indicate individual effect estimates. Positive values indicate higher fatigue scores. ANA and anti-Ro52 positivity were associated with higher total SSI; anti-SSB positivity with lower somatic fatigue; joint pain showed a trend toward higher somatic fatigue.

Among the evaluated predictors, only immunologic markers were associated with symptom scores. ANA and anti-Ro52 positivity were linked to higher SSI scores (*p* = 0.0277 and *p* = 0.0201, respectively), whereas anti-SSB positivity was associated with lower somatic fatigue (*p* = 0.0353). Joint pain displayed a borderline association with somatic fatigue (*p* = 0.0572). Hematologic and inflammatory markers, including leukocyte subsets, NLR, and PLR, showed no significant associations with any fatigue or sicca domains (Figure 4).

## 3. Discussion

In this cross-sectional study of 97 patients with primary Sjögren’s disease, we investigated the associations between hematological parameters, fatigue severity, and salivary gland histopathology, while also considering immunological markers and clinical manifestations. Fatigue, as assessed by the PROFAD-SSI questionnaire, was highly prevalent in our cohort, with median scores reflecting a substantial symptom burden. Although no strong correlations were observed between hematological parameters and fatigue scores, subtle trends emerged, particularly for leukocyte and platelet counts, which showed weak positive associations with total PROFAD scores. These findings suggest a possible, albeit modest, hematologic contribution to fatigue in Sjögren’s disease.

From an immunological perspective, ANA and anti-Ro52 positivity were associated with higher total SSI scores, whereas anti-SSB positivity was linked to lower somatic fatigue scores. Additionally, joint pain demonstrated a trend toward higher somatic fatigue. While these associations do not establish causality, they highlight potential pathways through which immune dysregulation and clinical manifestations may influence fatigue perception.

Our results align with the existing literature emphasizing the multifactorial nature of fatigue in Sjögren’s disease. Fatigue has been shown to be modulated by both peripheral and central mechanisms, with contributions from chronic immune activation, neuroinflammation, and psychosocial factors [17,38,39,40]. Importantly, our data suggest that routine hematological indices, although widely available and cost-effective, may have limited value as standalone biomarkers for fatigue severity. Nevertheless, the observed trends for leukocyte and platelet counts justify further exploration, particularly in larger, longitudinal cohorts where temporal relationships could be assessed.

Histopathological analysis of minor salivary gland biopsies did not show significant associations with fatigue severity. Focus score was unrelated to PROFAD or total PROFAD-SSI scores, and only a moderate inverse association with SSI was observed, indicating that greater lymphocytic infiltration did not correspond to increased subjective dryness. This dissociation between glandular inflammation and symptoms mirrors findings from earlier studies describing a mismatch between objective structural damage and subjective complaints in Sjögren’s disease [16,41,42]. Likewise, ultrasonographic grading of salivary glands (OMERACT scoring) showed no relationship with fatigue, supporting the concept that fatigue arises predominantly from systemic rather than glandular mechanisms [43].

Taken together, our findings reinforce the multifactorial nature of fatigue in Sjögren’s disease. Fatigue is thought to result from a complex interplay of peripheral inflammation, neuroimmune activation, autonomic dysregulation, and psychosocial factors, rather than from local glandular pathology or hematological abnormalities. This interpretation aligns with the current literature, which highlights the central role of neuroinflammatory pathways, cytokine-mediated sickness behavior, and psychological comorbidities in shaping fatigue expression [16,18,23].

The strengths of our study include a well-characterized patient cohort, the use of a validated multidimensional fatigue instrument, and the integration of clinical, immunological, and histopathological data. However, several limitations should be acknowledged. The cross-sectional design precludes causal inference, and the sample size limited the power for subgroup analyses, particularly for biopsy data. Treatment status at the time of blood sampling may also have influenced some clinical or laboratory parameters, as a proportion of patients were already receiving systemic therapy, whereas others were treatment-naive. Moreover, relevant covariates such as depression, sleep quality, and physical activity were not available for adjustment. Most hematologic parameters fell within normal laboratory ranges, which may have reduced their ability to discriminate between different levels of fatigue severity.

Future research should adopt longitudinal designs, incorporate comprehensive psychosocial and functional assessments, and explore whether combining hematological indices with immunological and clinical predictors can improve the identification of patients at risk for severe fatigue. Moreover, advanced analytical approaches, including cytokine profiling or metabolomics, may provide novel insights into the biological pathways linking immune dysregulation to fatigue in Sjögren’s disease.

## 4. Materials and Methods

This retrospective study included 97 patients diagnosed with primary Sjögren’s disease according to the 2016 ACR/EULAR classification criteria. These criteria were applied at the time of clinical evaluation and were used to confirm eligibility for inclusion. Data were collected at Sfanta Maria Clinical Hospital (Bucharest, Romania) between January 2024 and April 2025.

All patients underwent CBC, renal and liver function tests, and immunological evaluation including anti-Ro/SSA, anti-La/SSB antibodies, rheumatoid factor (RF), and antinuclear antibodies (ANA). Extra-glandular manifestations and associated comorbidities were systematically documented. Fatigue severity was evaluated using a validated patient-reported outcome measure, the Profile of Fatigue and Discomfort–Sicca Symptoms Inventory (PROFAD-SSI-SF).

The study was approved by the hospital’s institutional ethics committee, and informed consent was obtained from all participants.

Statistical analyses were performed using SPSS software, version 20.0 for Windows (SPSS Inc., Chicago, IL, USA). The distribution of continuous variables was assessed using the Kolmogorov–Smirnov and Shapiro–Wilk tests. Continuous data were expressed as mean ± standard deviation or mean ± standard error, as appropriate, while categorical variables were reported as absolute numbers and percentages.

Associations between hematological parameters and derived indices (NLR, PLR) were examined in relation to clinical manifestations and histopathological findings.

Comparisons between two independent groups were performed using the Mann–Whitney U test, given the predominantly non-normal distribution of the data. For comparisons across more than two groups, the Kruskal–Wallis test was applied. Correlation analyses between continuous variables were conducted using Spearman’s rho coefficients. Categorical variables were compared using the chi-square test or Fisher’s exact test, as appropriate. Multiple linear regression analyses were performed in three predefined models (hematologic, immunologic, and inflammatory) with fatigue scores as dependent variables. Variables with *p* < 0.10 in univariate analyses were entered into multivariable models, and only predictors remaining statistically significant (*p* < 0.05) or borderline significant (0.05 ≤ *p* < 0.10) were retained for the final summary. A two-tailed *p*-value < 0.05 was considered statistically significant.

Given the multifactorial nature of fatigue, additional covariates—including pain, dryness, mood, and sleep—were incorporated into the analysis to account for their potential influence on fatigue severity.

### 4.1. Inclusion and Exclusion Criteria

Patients were included if they were adults (≥18 years) with a diagnosis of Sjögren’s disease established by the presence of at least two of the following three criteria: (1) positive anti-SSA/Ro and/or anti-SSB/La antibodies, or a combination of positive rheumatoid factor (RF) and antinuclear antibody (ANA) titer ≥ 1:320; (2) minor salivary gland biopsy demonstrating focal lymphocytic sialadenitis with a focus score ≥ 1 per 4 mm^2^; (3) keratoconjunctivitis sicca, defined as an ocular staining score ≥ 3 using lissamine green.

Eligible patients were also required to have available data for complete blood count (CBC) and a validated assessment of fatigue, using the PROFAD-SSI questionnaire. Histopathological evaluation of minor salivary glands was also available for a subset of 41 patients.

Exclusion criteria included the presence of chronic or infectious diseases (including malignancies), use of medications known to affect salivary or hematologic function (such as anticholinergic, antidepressant, antihistaminic, diuretic, or neuroleptic agents), and diagnosis of connective tissue diseases that commonly overlap with Sjögren’s disease (e.g., rheumatoid arthritis, systemic lupus erythematosus, systemic sclerosis, or dermatomyositis). Additional exclusions were systemic vasculitis, prior head and neck radiotherapy, active hepatitis C infection, HIV/AIDS, sarcoidosis, amyloidosis, graft-versus-host disease, and IgG4-related disease.

These criteria are consistent with current classification systems and are intended to ensure a homogeneous study population while minimizing confounding factors that may influence sicca symptoms, hematologic parameters, or fatigue severity.

### 4.2. Blood Sampling and Patient Assessment

Blood samples were collected from the antecubital vein at 8:00 AM after an overnight fast. Treatment status at the time of sampling was recorded, distinguishing between treatment-naive patients and those already receiving systemic therapy. CBC parameters, including lymphocyte count, neutrophil count, platelet count, mean corpuscular volume (MCV), platelet distribution width (PDW), plateletcrit (PCT), hematocrit (HCT), mean platelet volume (MPV), and total white blood cell (WBC) count, were obtained. The neutrophil-to-lymphocyte ratio (NLR) and platelet-to-lymphocyte ratio (PLR) were calculated from these values. CBC analyses were performed using an automated hematology analyzer (Sysmex XN-1000; Sysmex Corporation, Kobe, Japan).

### 4.3. Biopsy Salivary Gland Protocol

Minor salivary gland biopsy (MSGB) had been performed as part of the diagnostic and classification workup. A total of 67 patients had available biopsy material. All specimens were processed using standard histopathological techniques.

Minor salivary gland biopsy (mSGB) was performed in the Otorhinolaryngology (ENT) Department under local anesthesia, with the patient in a supine position. After antiseptic preparation of the lower labial mucosa, 1% lidocaine without epinephrine was infiltrated locally. A chalazion clamp or similar device was applied to ensure a bloodless field and facilitate the identification of glandular tissue. A 1–1.5 cm linear or elliptical incision is made parallel to the vermilion border, avoiding the midline to reduce risk of sensory nerve injury. Blunt dissection is used to expose and excise 4–6 minor salivary glands, ensuring adequate tissue for histopathological analysis. Hemostasis is achieved with gentle pressure or cautery as needed. The wound is closed with absorbable sutures. A minimum of four surgically separated minor salivary glands were excised; in cases where glands were <2 mm in size, six were collected, following consensus guidelines [35,36,44,45].

Tissue samples were fixed in 10% neutral-buffered formalin, embedded in paraffin, and sectioned at a minimum of one level. If the initial histological evaluation was inconclusive—such as in borderline focus scores or insufficient surface area—up to two additional levels were examined at 200 µm intervals. Histopathological examination was performed using hematoxylin and eosin staining. The presence of focal lymphocytic sialadenitis (FLS)—defined as dense aggregates (foci) of ≥50 mononuclear cells in a periductal or perivascular distribution adjacent to preserved acini—was assessed. The focus score (FS) was calculated as the number of foci per 4 mm^2^ of total glandular surface area, which included both normal and atrophic/fibrotic tissue, in accordance with EULAR consensus recommendations. Glandular area was measured using a calibrated eyepiece or image analysis software.

Additional histological features, including acinar atrophy, ductal dilatation, fibrosis, NSCS, germinal center–like structures, and lymphoepithelial lesions, were recorded when present. All slides were reviewed by experienced pathologists, and scoring followed standardized procedures aligned with the EULAR Sjögren’s Histopathology Working Group guidelines [45].

## 5. Conclusions

This study is, to the best of our knowledge, the first to simultaneously evaluate hematological parameters, salivary gland histopathology, and fatigue severity in primary Sjögren’s disease using the PROFAD-SSI, a multidimensional and disease-specific assessment tool. Although routine hematologic indices showed no significant associations with fatigue, modest trends for leukocyte and platelet counts suggest potential relevance that warrants further investigation. Immunologic markers, including ANA, anti-Ro52, and anti-SSB, demonstrated measurable associations with patient-reported outcomes, reinforcing the contribution of autoimmune activity to symptom variability.

Histopathological findings, including focus score and structural glandular changes, were not related to fatigue severity, supporting the concept that fatigue in Sjögren’s disease is driven predominantly by systemic rather than glandular mechanisms. These results underscore the multifactorial nature of fatigue and the need to integrate hematologic, immunologic, clinical, and patient-reported measures when evaluating symptom burden.

Limitations include the cross-sectional design, incomplete biopsy data, and a modest sample size, which may have limited the power to identify subtle associations. Future studies should build on this work using larger, longitudinal cohorts and advanced analytical approaches to identify robust biomarkers and guide more targeted symptom management aimed at improving quality of life in primary Sjögren’s disease.

## Figures and Tables

**Table 1:** Demographic and clinical characteristics of the study population. Data are presented as mean ± standard deviation (SD) for continuous variables and as absolute numbers (percentage) for categorical variables. Percentages are calculated based on available data for each variable.

**Parameters**	**Results**
Age (years) mean ± SD	57.18 ± 14.13
Gender (F/M) *n* (%)	94 (96.9%)/3 (3.1%)
Age at diagnostic (years) mean ± SD	51.10 ± 14.33
Diagnostic interval (years) mean ± SD	1.59 ± 3.45
Smoking *n* (%)	14 (14.4%)
**Clinical manifestation**	
Dry mouth *n* (%)	90 (92.8%)
Dry eyes *n* (%)	90 (92.8%)
Joint pain *n* (%)	66 (68%)
Neurological manifestation *n* (%)	20 (20.6%)
Pulmonary manifestation *n* (%)	27 (27.8%)
**Treatment**	
Plaquenil *n* (%)	70 (72.2%)
Methotrexate *n* (%)	7 (7.2%)
Azathioprine *n* (%)	13 (13.4%)
Mycophenolate mofetil *n* (%)	7 (7.2%)
Glucocorticoids *n* (%)	35 (36.1%)
Treatment-naive at sampling *n* (%)	31 (32%)
Minor salivary gland biopsy *n* (%)	67 (69.1%)

**Table 2:** Correlations between hematological parameters and fatigue scores.

Hematological Parameter	Correlation with Total PROFAD	Correlation with Total SSI	Correlation with Total PROFAD-SSI
Hemoglobin (Hb)	0.15	0.05	0.05
Red blood cell count (RBC)	0.1	−0.01	0
Mean corpuscular volume (MCV)	−0.08	−0.08	−0.03
Leukocyte count	0.18	0.05	0.06
Neutrophile count	0.13	0.05	0.07
Lymphocyte count	0.13	0.04	0
Platelet count (PLT)	0.16	0.05	0.11
Neutrophile-to-lymphocyte ratio (NLR)	0	0.05	0.07
Platelet-to-lymphocyte ratio (PLR)	−0.03	0.01	0.06

**Table 3:** Selected clinical an immunologic predictor of somatic fatigue and total SSI scores in primary Sjögren’s disease.

Subgroup	Outcome	Effect (β ± SE or Median Δ)	*p*-Value
Anti-SSB positivity	Somatic fatigue	−0.51 ± 0.26	0.035
ANA positivity	Total SSI	↑ median (5.15 vs. 4.80)	0.028
AntiRo52 positivity	Total SSI	↑ median (4.95 vs. 4.80)	0.020
Joint pain	Somatic fatigue	↑ median (5.00 vs. 4.62)	0.057

↑ = increased/higher value.

**Table 4:** Correlations between focus score and symptom scores.

Histologic Variable	Fatigue Score	Spearman r	*p*-Value
Focus score	PROFAD	−0.09	0.67
Focus score	SSI	−0.48	0.021
Focus score	PROFAD-SSI	−0.36	0.093

## Data Availability

The data presented in this study are available on request from the corresponding author. The data are not publicly available due to privacy and ethical restrictions, as they contain confidential patient information.

## Abbreviations

The following abbreviations are used in this manuscript:

SjD	Sjögren’s disease
CBC	Complete blood cell count
RBC	Red blood cells
WBC	White blood cells
RDW	Red cell distribution width
MCV	Mean corpuscular volume
NLR	Neutrophile-to-lymphocyte ratio
PLR	Platelet-to-lymphocyte ratio
MLR	Monocyte-to-lymphocyte ratio
ILD	Interstitial lung disease
mSGB	Minor salivary gland biopsy
FLS	Lymphocytic sialadenitis
FS	Focus score
NSCS	Non-specific chronic sialadenitis
GCs	Germinal center-like structures
LESA	Lymphoepithelial lesions
